# miR-1296-5p decreases ERBB2 expression to inhibit the cell proliferation in ERBB2-positive breast cancer

**DOI:** 10.1186/s12935-017-0466-y

**Published:** 2017-10-24

**Authors:** Gang Chen, Mingfeng He, Yin Yin, Ting Yan, Wenfang Cheng, Zebo Huang, Lan Zhang, Huo Zhang, Ping Liu, Wei Zhu, Yichao Zhu

**Affiliations:** 1 Department of Oncology, The Affiliated Jiangning Hospital of Nanjing Medical University, Nanjing, 210029 People’s Republic of China; 2 Department of Oncology, First Affiliated Hospital of Nanjing Medical University, Nanjing, 210029 People’s Republic of China; 3 Department of Anesthesiology, First Affiliated Hospital of Nanjing Medical University, Nanjing, 210029 People’s Republic of China; 4 Department of Gynecology and Obstetrics, First Affiliated Hospital of Nanjing Medical University, Nanjing, 210029 China; 5 Safety Assessment and Research Center for Drug, Pesticide and Veterinary Drug of Jiangsu Province, Nanjing Medical University, Nanjing, 211166 People’s Republic of China; 6 Department of Gastroenterology, First Affiliated Hospital of Nanjing Medical University, Nanjing, 210029 People’s Republic of China; 7 Department of Physiology, Nanjing Medical University, Nanjing, 211166 People’s Republic of China; 8 State Key Laboratory of Reproductive Medicine, Nanjing Medical University, Nanjing, 211166 People’s Republic of China

**Keywords:** miR-1296-5p, ERBB2, Breast cancer, Proliferation, mTORC1

## Abstract

**Background:**

The tumor suppressive role of miR-1296 is observed in triple negative breast cancer (TNBC). However, the effect of miR-1296-5p in ERBB2-positive breast cancers remains obscure.

**Methods:**

Whether *ERBB2* was the target gene of the miR-1296-5p was predicted by online software, and determined by dual-luciferase activity assay. miR-1296-5p expression levels were determined in breast cancer samples (114 breast cancer tissues and 30 adjacent normal tissues) by using qRT-PCR. The effect of miR-1296-5p and inhibition of ERBB2/mTORC1 signaling on the downstream target was assessed by Western blot. SK-BR-3 and BT-474 breast cancer cell line was transfected with miR-1296-5p mimic after which cell proliferation and apoptosis were determined by the clonogenic assay and the flow cytometry system, respectively. In addition, the chemotherapeutic drug sensitivity of SK-BR-3 and BT-474 cells transfected with miR-1296-5p mimic were determined by MTT assay.

**Results:**

The luciferase assay carrying ERBB2 3′-untranslated region-based reporters expressed in SK-BR-3 and BT-474 cells suggested that *ERBB2* was the target gene of miR-1296-5p. MiR-1296-5p was significantly decreased in breast cancer tissues compared to adjacent normal tissues. Moreover, it was declined in ERBB2-positive breast cancer samples compared with that in ERBB2-negative breast cancer tissues. Overexpressed miR-1296-5p reduced its target protein level and mTORC1/S6 activation, inhibited the proliferation of ERBB2-positive breast cancer cells and sensitized these cells to cisplatin and 5-fluorouracil-induced apoptosis.

**Conclusions:**

Our findings suggest that miR-1296-5p is involved in the regulation of proliferation in breast cancer cells via targeting ERBB2/mTORC1 signaling pathway.

## Background

ERBB2 (or HER2) is a receptor tyrosine kinase, of which the amplification occurs in approximate a quarter of breast carcinomas and is identifies as particularly aggressive tumors [1–3]. Over-expressed ERBB2 couples to two major oncogenic pathways (RAS-ERK and PI3K-AKT) that promote cell survival, proliferation, migration and invasion [4, 5]. Although ERBB2-targetd therapy (herceptin or trastuzumab) has been successfully used to treat ERBB2-overexpressing tumors, patients’ drug resistance curbs its clinical widespread use [6–8]. There is an imperious need to develop new and efficient targets for therapeutic intervention of ERBB2-positive breast cancers.

MicroRNAs (miRNAs) have a wide range of ability to regulate cell survival, proliferation, differentiation, migration, invasion and metastasis of breast cancers [9–11]. MiR-1296 is shown to down-regulate the synthesis phase of the cell cycle in prostate cancers [12]. MiR-1296 also induces cell apoptosis via PIM1-STAT3 signaling pathway in cervical cancer [13]. MiR-1296 is association with chemotherapeutic resistance and self-renewal capability in breast cancers [14]. In TNBC cells and tissues, miR-1296 expression is significantly suppressed, and which sensitized TNBC cells to cisplatin treatment [15]. However, it is still largely unknown how specific miRNA functions affect ERBB2-positive breast cancers. Here, we demonstrated that miR-1296-5p decreases the expression level of ERBB2 to inhibit the cell proliferation in ERBB2-positive breast cancer.

## Methods

### Clinical samples

A total of 114 breast cancer patients recruited from the First Affiliated Hospital of Nanjing Medical University from 2013 to 2016 were included in this study. Samples consisted of 114 of breast cancer tissues and 30 of adjacent normal tissues. All tissues were histopathologically confirmed by a pathologist who selected tumor areas with higher tumor cell density that was suitable for RNA isolation and immunohistochemistry (IHC). All the samples were pathologically examined and stored in liquid nitrogen for miRNA analysis. Ethical approval for the study was granted by the Clinical Research Ethics Committee, Nanjing Medical University. Written informed consent was taken from each participant.

### Cell culture

SK-BR-3 human breast cancer cell line was purchased from the Cell Bank of Shanghai (Shanghai, China). BT-474 breast cancer cell line was kindly gifted from Dr. Tiansong Xia (First Affiliated Hospital of Nanjing Medical University). Cells were cultured in RPMI 1640 or DMEM medium, supplemented with 10% fetal bovine serum (Hyclone, Logan, UT, USA), at 37 °C in a humidified atmosphere with 5% CO_2_.

### Dual-luciferase activity assay

The 3′-UTR of human ERBB2 containing the putative target site for the miR-1296-5p was chemically synthesized and inserted at the *Xba*I site, immediately downstream of the luciferase gene in the pGL3-control vector (Promega, Madison, WI) by Integrated Biotech Solutions Co., Ltd (Shanghai, China), respectively. Twenty-four hours before transfection, cells were plated into 24-well plates (1.5 × 10^5^ cells/well). PGL3-ERBB2-3′-UTR (200 ng) and pRL-TK (80 ng, Promega) were transfected in combination with 60 pmol miR-1296-5p mimic or miRNA mimic control using Lipofectamine 2000 (invitrogen). Luciferase activity was measured 24 h after transfection using the dual luciferase reporter assay system (Promega). Firefly luciferase activity was normalized to renilla luciferase activity for each transfected well. Three independent experiments were performed in triplicate.

### Western blot analysis

SK-BR-3 or BT-474 cells were plated in 6-well plates (6 × 10^5^ cells/well). Seventy-two hours after the transfection of miR-1296-5p mimic or miRNA mimic control, cells were harvested and homogenized with lysis buffer. Total protein was separated by 10% denaturing SDS–polyacrylamide gel electrophoresis. Western blot analysis was performed as described [16]. The primary antibodies for ERBB2, S6, Phospho-S6, beta-actin, and GAPDH were purchased from cell signaling technology (Danvers, MA). The expression levels of indicated proteins were normalized to GAPDH or beta-actin.

### Quantitative real-time PCR analysis for miRNA

Breast cancer tissues and cells were isolated with Trizol reagent (Invitrogen, Carlsbad, CA). MiRNA fraction was purified using a mirVana™ miRNA isolation kit (Ambion, Austin, TX). The concentration and purity of the RNA samples were determined spectroscopically. The SYBR and U6 gene were used for detecting the gene amplification and normalizing the each sample, respectively. The primers of reverse transcription and polymerase chain reaction were purchased from RiboBio Co., Ltd (Guangzhou, China). QRT-PCR was performed according to the protocol of primer sets. PCR product amplification was detected by the level of fluorescence emitted by SYBR Green (SYBR® Premix Ex Taq™ II, TaKaRa) which intercalated into double stranded DNA [17]. The ΔCt method was used for miRNA expression analysis of biopsy specimens. The cycle number at the threshold level of fluorescence (Ct) for each sample was determined, and then calculating the ΔCt value. The ΔCt value was the subtraction between the Ct value of miR-1296-5p and the Ct value of U6: ΔCt = Ct (miR-1296-5p) − Ct (U6). The fold-change for miRNA compared to each controls was calculated using the 2^−ΔΔCt^ method. PCR was performed in triplicate.

### Clonogenic assay

SK-BR-3 or BT-474 cells were transfected with miR-1296-5p mimic or miRNA mimic control and plated into 6-well plates at a density of 1000 cells/well, incubated at routine culture condition for 2 weeks, fixed and stained with crystal violet. The number of colonies were counted under a microscope from three independent replicates.

### Apoptosis assay

SK-BR-3 cells were plated into 6-well plates (6 × 10^5^ cells/well). Forty-eight hours after the transfection of miR-1296-5p mimic or miRNA mimic control, flow cytometry was used to detect apoptosis of the transfected SK-BR-3 cells by determining the relative amount of annexin V-FITC-positive and propidium iodide (PI)-negative cells. Annexin V-FITC and PI were purchased from BD biosciences (San Jose, CA).

### RhoA and Rac1 activation assay

For RhoA and Rac1 activation assays, breast cancer cells were plated into 35 mm dishes and transfected with miR-1296-5p mimic or miRNA mimic control. The experiments were then performed according to the manufacturer’s protocol (Cytoskeleton Inc., Denver, CO, USA).

### In vitro drug sensitivity assay

SK-BR-3 or BT-474 human breast cancer cells were plated into 35 mm dishes (6 × 10^5^ cells/dish), 100 nmol/L of the miR-1296-5p mimic or 100 nmol/L miRNA mimic control were transfected in SK-BR-3 cells, using lipofectamine 2000 (Invitrogen, Long Island, NY, USA). The miR-1296-5p mimic, miRNA mimic control were chemically synthesized by Shanghai GenePharma Company (Shanghai, China). Twenty-four hours after transfection cells were seeded into 96-well plates (5 × 10^3^ cells/well). Forty-eight hours after the drug treatment, cell viability was assessed by 3-(4,5-dimethylthiazol-2-yl)-2,5-diphenyl-tetrazolium bromide (MTT, Beyotime Biotechnology, Nantong, China) assay. The absorbance at 490 nm of each well was read on a spectrophotometer. The concentration at which drugs produced 50% inhibition of growth (IC50) was estimated by the relative survival curve. Three independent experiments were performed in quadruplicate.

### Statistical analysis

Each experiment was repeated at least 3 times. Numerical data were presented as mean ± SD. The difference between means was analyzed with Student’s t test. All statistical analyses were performed using SPSS 13.0 software (Chicago, IL). Differences were considered significant when *p* < 0.05.

## Results

### ERBB2 as the target genes of miR-1296-5p

**Fig. 1:**
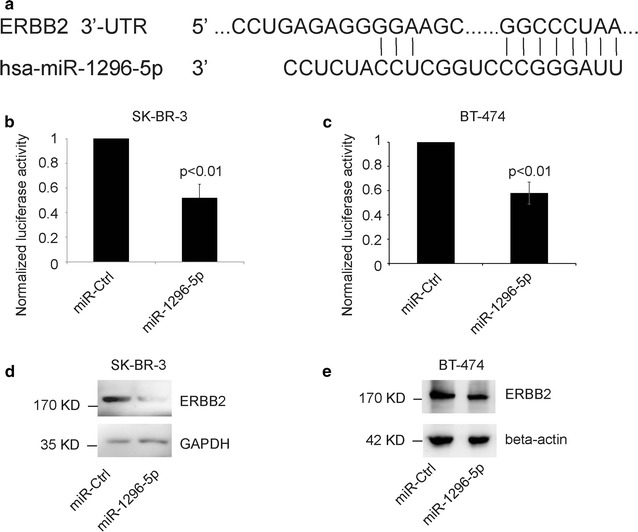
ERBB2 as target of miR-1296-5p. **a** The seed sequence of miR-1296-5p is complementary to the 3′-UTR of ERBB2. **b**, **c** Luciferase assay showing reduction in reporter activity (relative luciferase units) after co-transfection of ERBB2-3′UTR with miR-1296-5p in SK-BR-3 or BT-474 cells. **d**, **e** Western blot analysis showing suppression of ERBB2 protein levels in SK-BR-3 or BT-474 cells after miR-1296-5p overexpression

TargetScanHuman (http://www.targetscan.org) suspects that *ERBB2* is a target gene of the miR-1296-5p (Fig. 1a). To determine whether *ERBB2* is the target gene of miR-1296-5p, the luciferase reporter vectors carrying the putative *ERBB2* 3′-UTR target site for the miR-1296-5p (pGL3-ERBB2-3′-UTR) were constructed and then transfected into SK-BR-3 or BT-474 cells. The relative luciferase activity was significant decreased in miR-1296-5p-overexpressing cells than that in control cells (Fig. 1b, c). Next, the miR-1296-5p mimics were transfected into SK-BR-3 or BT-474 cells and the expression levels of ERBB2 were examined by Western blot. We found that the expression of ERBB2 in miR-1296-5p-transfected cells was extremely depressed than that in control cells (Fig. 1d, e). Taken together, these results indicate that *ERBB2* is a target gene of the miR-1296-5p.

### MiR-1296-5p was down-regulated in breast cancer tissues and ERBB2-positive tissues

**Fig. 2:**
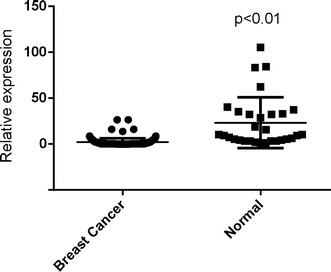
MiR-1296-5p is down-regulated in breast cancer tissues. The miR-1296-5p expression is suppressed in a majority of breast cancer samples (n = 114) when compared to normal breast samples (n = 30). The miRNA relative expression levels were normalized to the average value of breast cancer samples

**Fig. 3:**
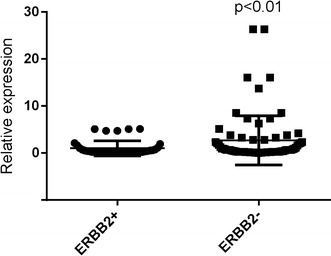
MiR-1296-5p is down-regulated in ERBB2-positive breast cancer tissues. The miR-1296-5p expression is suppressed in a majority of ERBB2-positive breast cancer samples (n = 40) when compared to ERBB2-negative breast cancer samples (n = 74). The miRNA relative expression levels were normalized to the average value of ERBB2-positive breast cancer samples

To determine whether miR-1296-5p expression was associated with breast cancer, miR-1296-5p expressions in breast cancer tissues including 114 breast cancer tissues and 30 non-tumor adjacent normal tissues were examined. The expression level of miR-1296-5p was significantly declined in breast cancer samples compared with the non-tumor adjacent normal tissues (Fig. 2). Meanwhile, miR-1296-5p was significantly down-regulated in human ERBB2-positive breast cancer tissues compared with ERBB2-negative breast cancer tissues (Fig. 3).

### MiR-1296-5p suppressed mTORC1 signaling in breast cancer cells

**Fig. 4:**
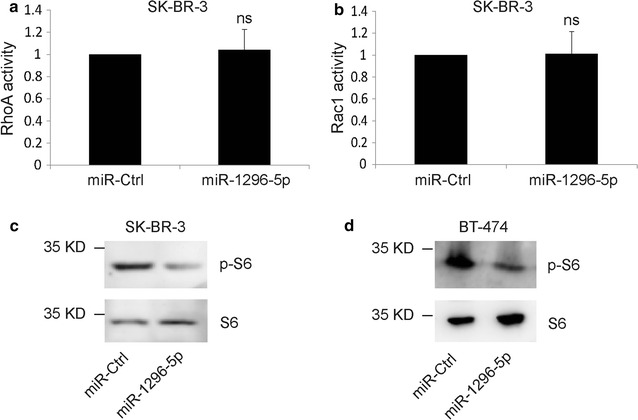
MiR-1296-5p suppresses mTORC1 signaling. **a**, **b** miR-1296-5p overexpression did not alter the activation of RhoA and Rac1 in SK-BR-3 breast cancer cells. The RhoA or Rac1 relative active levels were normalized to the average value of SK-BR-3 cells transfected with control miRNA. ns, no significance. **c**, **d** miR-1296-5p overexpression suppresses the phosphorylation of S6 in SK-BR-3 or BT-474 breast cancer cells

Previous studies reported that ERBB2 induction of the invasion and migration of breast cancer cells required activation of the small GTPases and mTOR signaling [18, 19]. We examined the activation of RhoA, Rac1 and mTORC1 signaling in miR-1296-5p-transfected cells and the control cells. We found that the activation of RhoA and Rac1 were not significantly changed in miR-1296-5p-transfected cells compared to those in control cells (Fig. 4a, b). Interestingly, the phosphorylation of S6, the direct downstream of mTORC1 signaling, was obviously down-regulated in miR-1296-5p-transfected SK-BR-3 or BT-474 cells (Fig. 4c, d). These results indicate that miR-1296-5p mediates the mTORC1 signaling, but not Rho signaling, in breast cancer cell lines.

### MiR-1296-5p inhibited cell proliferation of breast cancer cells

**Fig. 5:**
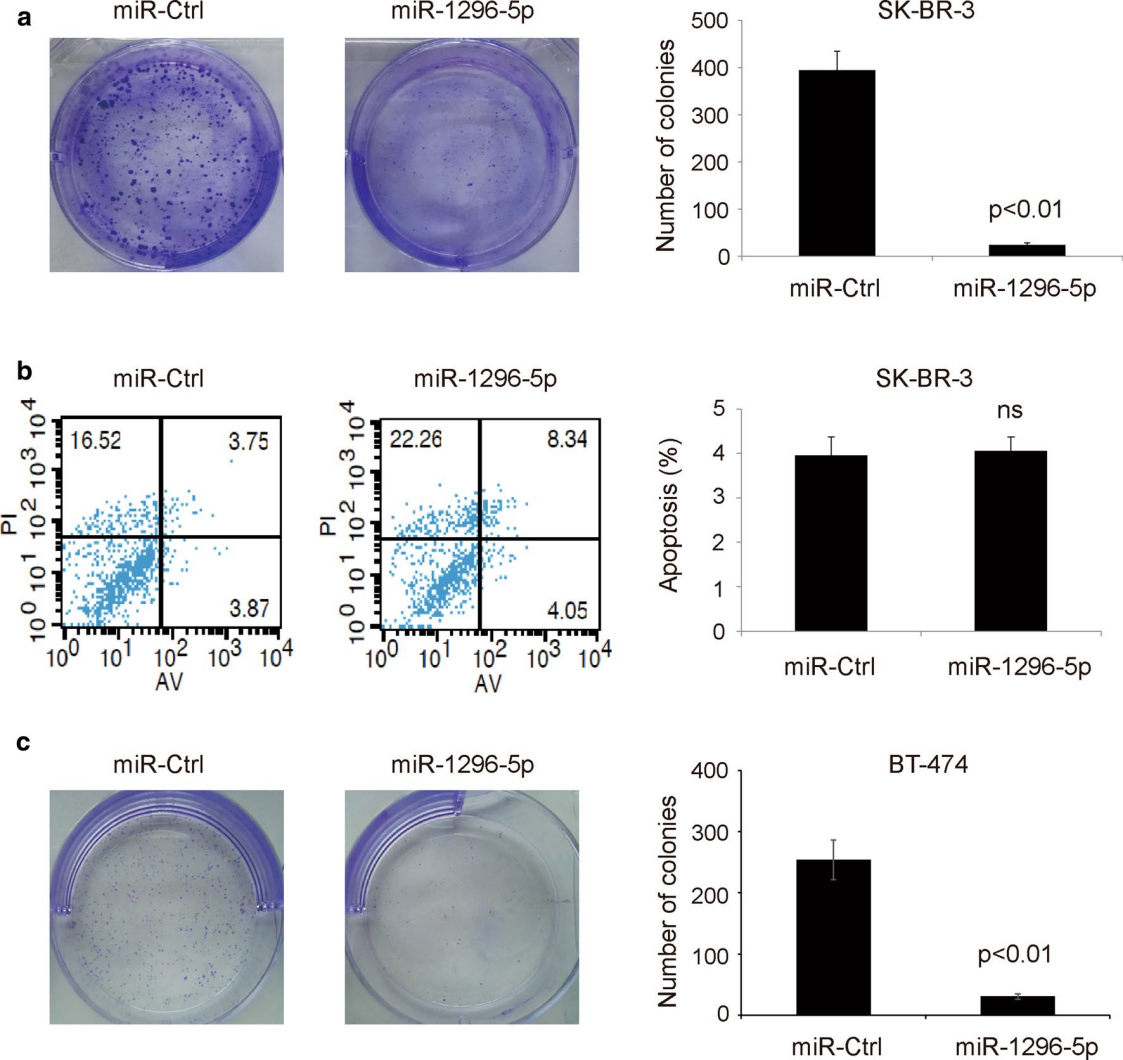
MiR-1296-5p suppresses the colony formation ability of ERBB2-positive breast cancer cells. **a**, **c** miR-1296-5p overexpression reduced the colony formation ability of SK-BR-3 or BT-474 breast cancer cells when compared to control miRNA-expressing cells. **b** miR-1296-5p overexpression did not alter the apoptosis of SK-BR-3 breast cancer cells when compared to control miRNA-expressing cells. *ns* no significance

Clonogenic assay revealed that SK-BR-3 or BT-474 cells transfected with miR-1296-5p were shown significantly reduced clone formation compared with the control cells (Fig. 5a, c). Moreover, the overexpression of miR-1296-5p did not alter the apoptosis of SK-BR-3 cells (Fig. 5b). These results demonstrate that miR-1296-5p inhibits cell proliferation of breast cancer cell line.

### MiR-1296-5p turned SK-BR-3 cells more vulnerable to the apoptosis induced by cisplatin and 5-fluorouracil

**Fig. 6:**
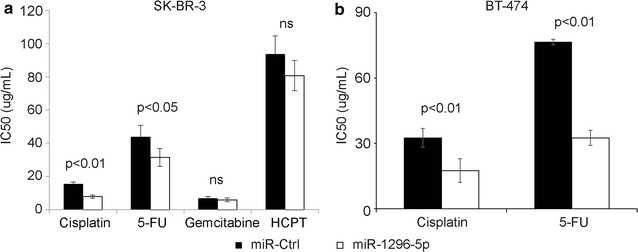
MiR-1296-5p sensitizes cells to chemotherapy drugs. **a**, **b** Overexpression of miR-1296 in SK-BR-3 or BT-474 breast cancer cells significantly sensitized cells to cisplatin and 5-fluorouracil (5-FU) treatment as compared to control miRNA. Cells were treated with cisplatin 24 h following miR-1296-5p transfection and the cell viability was assessed after 48 h of cisplatin treatment. 5-FU, 5-fluorouracil. HCPT, hydroxy camptothecin. *ns* no significance

Since the miR-1296-5p inhibits cell proliferation of breast cancer cell line, a hypothesis is proposed that miR-1296-5p might evoke the apoptosis of ERBB2-positive breast cancer cells by weakening drug resistance. Thus, drug-induced apoptosis in chemotherapy was evaluated through transfecting SK-BR-3 or BT-474 cells with miR-1296-5p. According to MTT assay, cells transfected with miR-1296-5p mimic exhibited greatly decreased resistance to cisplatin and 5-fluorouracil (5-FU) compared with the control cells (Fig. 6a, b). However, miR-1296-5p did not turn the vulnerable to the apoptosis induced Gemcitabine and HCPT, respectively (Fig. 6a). These results demonstrate that miR-1296-5p turns breast cancer cells more vulnerable to the apoptosis induced by cisplatin and 5-FU.

## Discussion

In this study, we find that miR-1296-5p expression is suppressed in a majority of breast cancer samples when compared to normal breast samples. Previous study has showed that the expression level of miR-1296 is decreased in prostate cancer and triple negative breast cancer (TNBC) samples [12, 15], suggesting that it is a tumor-suppressor gene in these cancers. MiRNA-next generation sequencing (NGS) analysis reveals miR-1296 is associated with chemotherapeutic resistance and self-renewal capability in breast cancers [14]. MiRNA microarray analyses demonstrate that miR-1296 affects PIM1-STAT3 pathway and subsequently induces cell apoptosis in cervical cancer [13]. These studies show that the function of miR-1296 as a tumor-suppressor might be more conflicting. In the other diseases, miR-1296 is putative negative regulators of XAF1 in lymphoblastoid cell lines (LCLs) during long-term cell culture, suggesting that miR-1296 is participated in the terminal immortalization of LCLs [20]. The expression of miR-1296 is increased in sera of chronic congestive heart failure (CHF) patients, which may be a potential prognostic marker in CHF [21]. Here, we found that miR-1296-5p was decreased in ERBB2-positive breast cancer samples when compared to ERBB2-negative breast cancer samples, suggesting that miR-1296-5p might be a tumor-suppressor gene in ERBB2-positive breast cancer.

It has been reported that miR-1296 overexpression significantly inhibit the expression of cyclin D1 and cell proliferation of TNBC cell lines [15]. In human prostate carcinoma PC3 cells, knockdown of miR-1296 increases both minichromosome maintenance 2 (MCM2) mRNA and protein [12]. To the contrary, miR-1296 overexpression decreases the expression of MCM2 mRNA and protein, and shortens the synthesis phase of the cell cycle [12]. Here, we found that miR–1296-5p inhibits the proliferation of ERBB2-positive breast cancer cells by decreasing ERBB2 expression. ERBB2 can activate mTORC1/p70S6K signaling in human breast cell lines and breast cancers [22]. Consistent with the report, we found that miR-1296-5p suppressed ERBB2 expression and mTORC1 signaling, subsequently inhibited the proliferation of ERBB2-positive breast cancer cells.

MiR-1296 expression sensitized TNBC cells to cisplatin treatment [15]. We also found that miR-1296-5p sensitized SK-BR-3 cells to cisplatin and 5-FU-induced apoptosis. Therefore, it is possible that miR-1296-5p may modulate the resistance of chemotherapeutic agents, and serve as novel targets for antitumor therapies.

## Conclusions

In summary, this study demonstrate that miR-1296-5p might be involved in the regulation of proliferation in human breast cancer cells via targeting ERBB2/mTORC1 signaling pathway. Therefore, miR-1296-5p is a tumor suppressor in breast cancer and a potential clinical diagnostic marker and therapeutic target for human breast cancer.

## Abbreviations

TNBC: triple negative breast cancer
RTK: receptor tyrosine kinase
IHC: immunohistochemistry
mTOR: mammalian target of rapamycin
qRT-PCR: quantitative real-time PCR
5-FU: 5-fluorouracil
HCPT: hydroxy camptothecin
NGS: next generation sequencing
LCL: lymphoblastoid cell line
CHF: congestive heart failure
MCM2: minichromosome maintenance 2
MTT: 3-(4,5-dimethylthiazol-2-yl)-2,5-diphenyl-tetrazolium bromide
IC50: 50% inhibition of growth
